# Bioprospecting of wild type ethanologenic yeast for ethanol fuel production from wastewater-grown microalgae

**DOI:** 10.1186/s13068-021-01925-x

**Published:** 2021-04-09

**Authors:** Enrique Romero-Frasca, Sharon B. Velasquez-Orta, Viviana Escobar-Sánchez, Raunel Tinoco-Valencia, María Teresa Orta Ledesma

**Affiliations:** 1 Instituto de Ingeniería, Coordinación de Ingeniería Ambiental, Universidad Nacional Autónoma de México, Apartado Postal 70-472, Coyoacán, 04510 Ciudad de México, México; 2 School of Engineering, Merz Court, Newcastle University, Newcastle upon Tyne, UK; 3 Laboratorio de Biología Molecular Y Genómica, Facultad de Ciencias, Universidad Nacional Autónoma de México, Ciudad de México, México; 4 Instituto de Biotecnología, Unidad de Escalamiento Y Planta Piloto, Universidad Nacional Autónoma de México, Cuernavaca, Morelos, México

**Keywords:** *Candida* sp., Fermentation, Hydrolysis, Municipal wastewater, Microalgae, *Scenedesmus* sp

## Abstract

**Background:**

Wild-type yeasts have been successfully used to obtain food products, yet their full potential as fermenting microorganisms for large-scale ethanol fuel production has to be determined. In this study, wild-type ethanologenic yeasts isolated from a secondary effluent were assessed for their capability to ferment saccharified microalgae sugars.

**Results:**

Yeast species in wastewater were identified sequencing the Internal Transcribed Spacers 1 and 2 regions of the ribosomal cluster. Concurrently, microalgae biomass sugars were saccharified via acid hydrolysis, producing 5.0 ± 0.3 g L^−1^ of fermentable sugars. Glucose consumption and ethanol production of yeasts in hydrolyzed-microalgae liquor were tested at different initial sugar concentrations and fermentation time. The predominant ethanologenic yeast species was identified as *Candida* sp., and glucose consumption for this strain and *S. cerevisiae* achieved 75% and 87% of the initial concentration at optimal conditions, respectively. Relatively similar ethanol yields were determined for both species, achieving 0.45 ± 0.05 (*S. cerevisiae*) and 0.46 ± 0.05 g ethanol per g glucose (*Candida* sp.).

**Conclusion:**

Overall, the results provide a first insight of the fermentation capacities of specific wild-type *Candida* species, and their potential role in ethanol industries seeking to improve their cost-efficiency. 
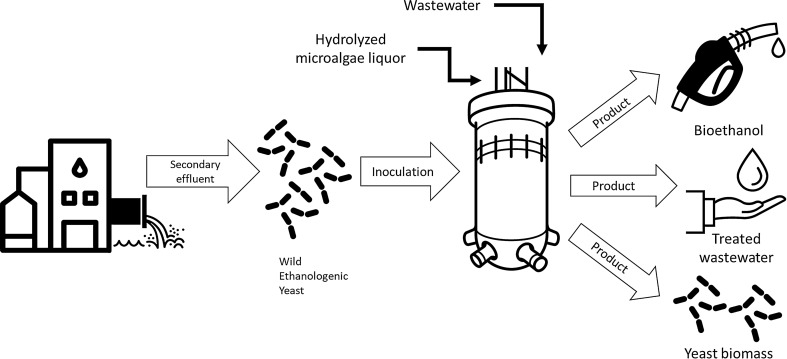

**Supplementary Information:**

The online version contains supplementary material available at 10.1186/s13068-021-01925-x.

## Background

Rapid socioeconomic growth around the world has produced an unquestionable increase in global energy demand. By 2020, more than 81% of the world total primary energy supply derives from nonrenewable fuel resources [1]. In order to reduce the dependency on fossil fuels, and the environmental issues associated with these, renewable fuel resources have been widely studied, and encouraged to fulfill the global energy demand in the near future [2–4]. Even though 12% [5] of the global energy demand is supplied by renewable energies (e.g., liquid biofuels, solar energy, and hydroelectric power plants) as of 2020, its overall share is expected to reach over 60% in the following years [6, 7].

Ethanol fuel, or simply ethanol, is one of the most demanded and important biofuels worldwide. In contrast to gasoline, ethanol is considered a cleaner alternative due to its high biodegradability, low greenhouse gas emissions, up to 96% less than fossil fuels, and null toxicity [8–11]. In addition, it is miscible with gasoline and can be used as an oxygenated portion in spark-ignition engines to reduce CO_2_ emissions [12, 13]. Currently, ethanol fuel is mostly produced from either edible-crops, also called first-generation ethanol, or agricultural residues, known as second-generation ethanol [11, 14, 15]. Nonetheless, the consolidation of these feedstocks in ethanol fuel industries are on hold due to socioeconomic and environmental impairments, expensive pretreatment, and complicated processing technologies [9, 10, 16]. Hence, further development of third-generation biomass sources, such as microalgae biomass, is deemed important as it is a promising feedstock due to its high growth rates, rapid carbohydrates accumulation, simpler sugar profile, one-step hydrolysis, and the ability to harvest nutrients from wastewater [4, 17–19]. Despite the indisputable potential of microalgae biomass, major drawbacks still need to be overcome to produce large volumes of ethanol fuel from microalgal biomass [20, 21].

Industrial ethanol production depends mostly on microbial activity, particularly that of yeasts. These microorganisms have produced valuable goods for centuries and one species, *Saccharomyces cerevisiae*, has a long tradition in the ethanol industry [22, 23]. More novel microbial alternatives have been studied in order to obtain a more economical and spontaneous ethanol fuel production in large-scale fermentation processes [24–26]. The usage of wild-type or non-conventional yeasts has gained interest in recent years due to several industrially relevant traits such as consumption of complex, inexpensive media, tolerance against stress and fermentation inhibitors [27]. Previous studies by Holt et al. [28] and Serra Colomer et al. [29] reported brewery-relevant properties of a broad range of wild yeast strains, belonging to *Brettanomyces, Cyberlindnera and Pichia*, genres, such as enhancement of aroma and flavor profiles when co-cultured with *S. cerevisiae*. On the other hand, our research group reported the dominance of ethanologenic yeast over microalgae when treating wastewater under heterotrophic conditions [30], which would allow integrated ethanol fuel production with wastewater treatment in a biorefinery-like process. Even if next-generation sequencing technologies and molecular engineering tools offer the possibility of mapping free-living yeast mechanisms for substrate assimilation and high-value bioproducts accumulation, wild-type yeasts’ full potential as an alternative to genetically modified or conventional yeasts in the ethanol fuel industry is yet to be determined. The aim of this study was to isolate and identify potential ethanologenic yeast in municipal wastewater secondary effluent to produce ethanol using saccharified microalgae sugars and synthetic wastewater.

## Results and discussion

A stepwise study starting from the culturing, harvesting, and pretreatment of *Scenedesmus* sp. predominant cultures to the alcoholic fermentation was performed. After harvesting, a total concentration of 100.0 ± 2.0 g L^−1^ of microalgae biomass was obtained prior to the acid hydrolysis step. Analyses in this study revealed that ethanologenic wild-type yeast thrive in municipal wastewater and certain species were able to effectively produce ethanol using hydrolyzed-microalgae liquor.

### Acid hydrolysis

Glucose was determined as the predominant monosaccharide, accounting for 4.15 g L^−1^ (83.7 ± 0.4%) of the total extracted sugar content in the resulting hydrolysate liquor. Although other monosaccharides (i.e., maltose and xylose) were also detected, these sugars only reached a total concentration of 0.35 (7.0 ± 0.3%) and 0.5 g L^−1^ (10 ± 0.5%) in the liquor, respectively. As for the total sugar content in the residual slurry, this resulted in less than 0.01 g L^−1^. Significant differences were observed between the total sugar content in untreated microalgae biomass and the hydrolysate liquor (*p* ≤ 0.05), confirming the importance of microalgae pretreatment for biofuels production.

A comparison of the hydrolysate liquor total extracted sugars and saccharification yield with literature is shown in Table 1. To start with, the final sugar concentration in the hydrolysate liquor was different in comparison to other studies. This is due to the total carbohydrate content of the microalgae biomass used in this study. Even though previous studies have used microalgae strains with a carbohydrate concentration exceeding 40% of the dry biomass, most of them employ axenic or single strain cultures which are costly and energy intensive when not performed in lab-scale [31, 32]. Moreover, such monocultures are at high risk of contamination that results in capital and product losses during manufacturing [33]. Thus, microalgae consortiums represent a possible cost reduction in the downstream processing of biomass as culture monitoring for contamination is relatively minimal and an enhanced co-processing of bioproducts could be achieved.

**Table 1:** Comparison of total sugar extraction and saccharification yields obtained with similar studies

Microalgae	Initial biomass (g L^−1^)	Carbohydrates biomass (%^a^)	Sulfuric acid (%v/V)	Temperature (°C)	Time (min)	Sugar extraction (%Ex) (%)	Saccharification (%Sa) (%)	Sugar content^b^ (g L^−1^)	Reference
*Scenedesmus* sp. Consortium	100	7.0 ± 0.9	5.0	90	120	71.5 ± 0.3	70.2 ± 0.2	5.0 ± 0.3	This study
*Chlorella* sp. ABC-001	50	39.1	3.0	90	150	86.9	71.5	17.0	Seon et al. [37]
*Scenedesmus* *Obliquus*	50	23.0 ± 2.0	3.0	120	30	90.0 ± 0.3	64.4 ± 0.7	20.7 ± 2.1	de Farias Silva et al. [11]
*Scenedesmus* sp. Consortium	100	ND	5.0	80–90	120	ND	49.1	16.6	Castro et al. [38]
*Scenedesmus* *Obliquus*	50	14.6	5.0	120	30	72.3	46.2	3.2	Miranda et al. [39]

^a^% of dry cell weight

^b^Sugar content estimates are measured in grams of sugar per liter of hydrolysate liquor

*ND* not determined

Additionally, total sugar extraction and saccharification yields were above the average when compared to the literature (Table 2). Even though only one study showed a higher sugar extraction yield, this appears to be attributed to the higher temperatures employed. For instance, not only de Farias Silva et al. [11] total sugar extraction exceed 90% of the carbohydrate content measured for the untreated biomass, but a temperature above 120 °C was achieved through autoclaving. Diluted acid in combination with autoclaving is one of the most common methods for microalgae feedstock pretreatment due to its relatively simple operation and relatively high sugar extraction yield [4, 34]. However, autoclaving is a costly and energy-intensive process, which is not suitable for all cell types and requires high temperatures in order to cause cell lysis [35]. Consequently, extracted sugars could be subject to dehydrations (i.e., thermal degradation) when reaction times are not precisely controlled and fermentation inhibitors, including but not limited to acetic acid, formic acid, hydroxymethylfurfurals (HMFs) and other furfurals, could be produced [2, 13, 36]. Hence, the above-average total sugar extraction (%Ex) and saccharification yield (%Sa) yields observed in this study are more likely due to longer reaction times and temperatures below 120 °C.

**Table 2:** Comparison of fermentation assays optimal conditions with similar studies

Microorganism	Initial sugar concentration (g L^−1^)	Sugar consumption (g L^−1^)	Net sugar consumption (%)	Ethanol (g L^−1^)	Ethanol yield (%)	Ethanol productivity (g L^−1^ h^−1^)	Acetic acid (g L^−1^)	Reference
*Candida* sp.	5.0 ± 0.3	3.6 ± 0.2	75.2 ± 0.2	2.2 ± 0.1	85.8 ± 0.01	0.150 ± 0.01	1.38 ± 0.05	This study
*Saccharomyces cerevisiae* S288C	5.0 ± 0.3	4.2 ± 0.4	87.2 ± 0.4	2.1 ± 0.1	81.7 ± 0.02	0.129 ± 0.06	1.45 ± 0.10	
*Clostridium acetobutylicum* (MTCC India)	31.45	ND	ND	1.0	6.2	0.0083	ND	Kallarakkal et al. [48]
*S. cerevisiae KL17*	13.5	8.5	63	3.5	50.7	0.318	ND	Seon et al. [37]
*S. cerevisiae* (Cameo S.p.A™)	13.0	12.1 ± 0.6	92.6 ± 4.4	4.9 ± 0.1	75.0	0.383 ± 0.10	0.69 ± 0.06	de Farias Silva et al. [11]
*Clostridium saccharoperbutylacetonicum N1–4*	16.6	ND	ND	0.5 ± 0.1	6.2 ± 0.02	0.030 ± 0.06	ND	Castro et al. [38]

*ND* not determined

### Yeast identification and characteristics

**Fig. 1:**
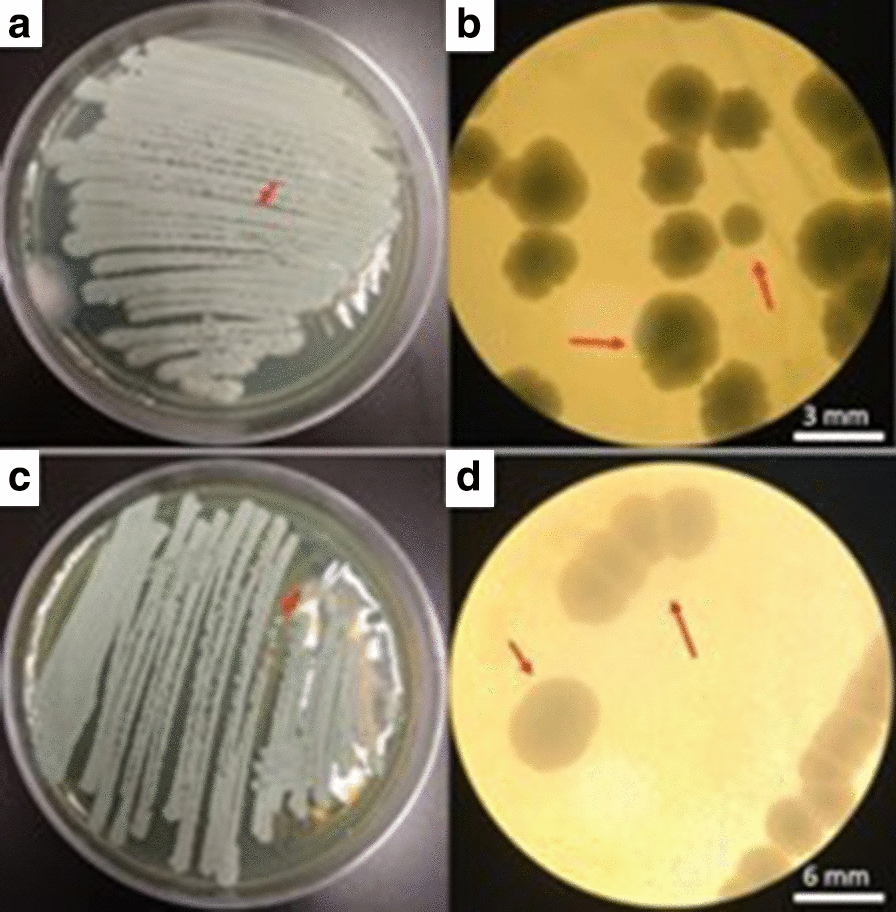
Morphological analysis of *Candida* sp. (**a**, **b**) and *Pichia* sp. (**c**, **d**) using a stereoscopic microscope

The purification and colonies’ morphological observations are shown in Fig. 1. Two colonial morphologies were observed, where one exhibited an irregular form, raised-type elevation, and undulated margin (Fig. 1b) while the other colony showed a nearly circular form, raised-type elevation, and entire margin (Fig. 1d). The yeast strains were identified as *Lindnera* sp. (anamorph of *Candida* sp.) and *Pichia* sp. through rDNA sequencing. *Candida* sp., as most species belonging to the *Candida* taxonomic genre, is an extremely heterogeneous unicellular species and its use in biotechnological and pharmaceutical industries has steadily increased in recent years [40]. For instance, certain *Candida* species have been used as forage or fodder yeast for livestock due to its high content in valuable and easily processed single-celled proteins or SCP [41, 42]. However, to employ *Candida* sp. as a fermenting microorganism for ethanol production, studies are still needed as it has not yet been fully characterized.

**Fig. 2:**
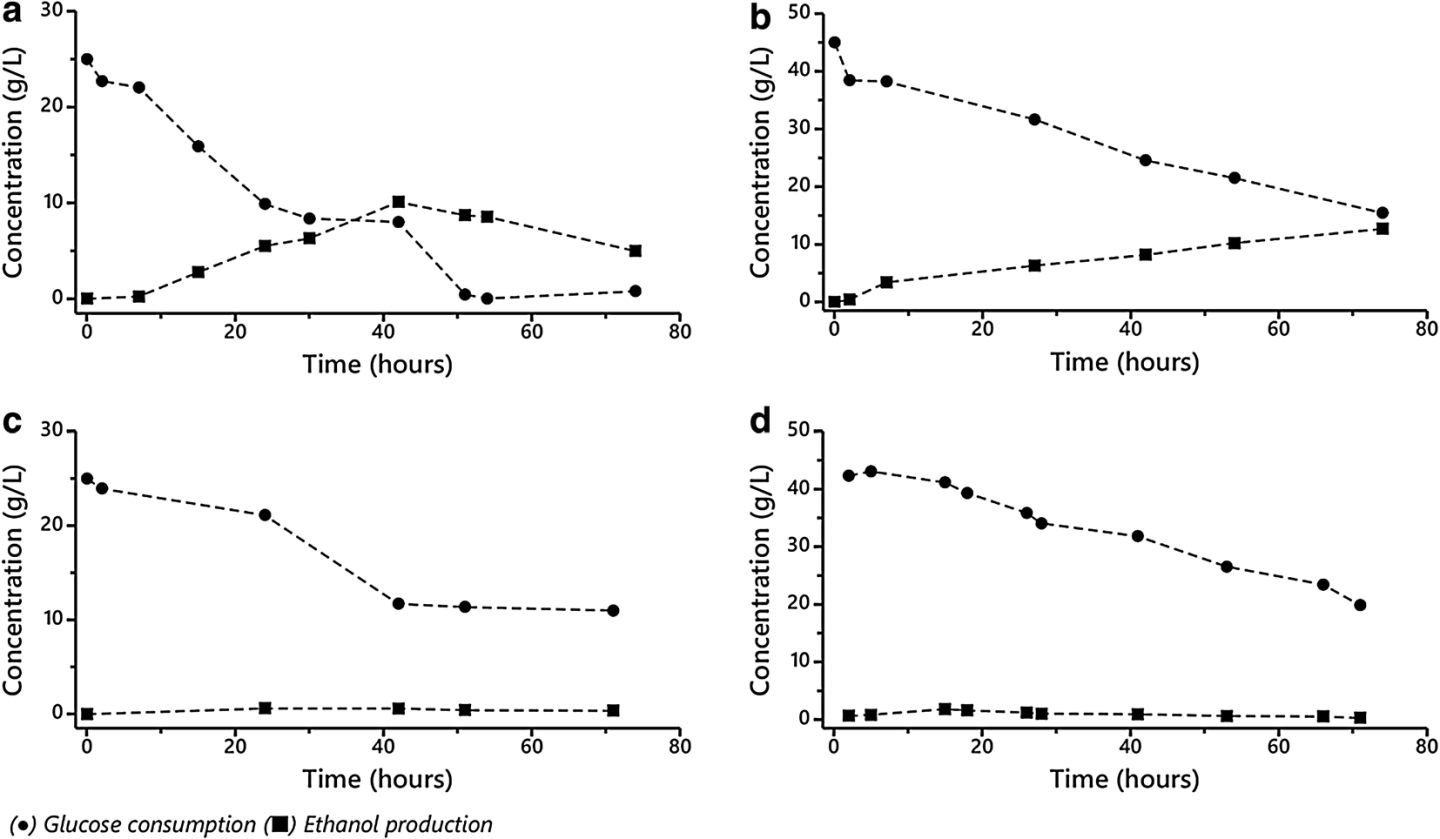
Glucose consumption and ethanol production for yeast isolates. *Candida* sp.: **a** 25 g L^−1^ dextrose-enriched filtered wastewater; **b** 45 g L^−1^ dextrose-enriched filtered wastewater. *Pichia* sp.: **c** 25 g L^−1^ dextrose-enriched filtered wastewater; **d** 45 g L^−1^ dextrose-enriched filtered wastewater. No replicates

Concurrently, *Pichia* sp. has a cosmopolitan distribution in nature, and it is often found in spoiled foods and fruit juices. This strain has been cataloged as clinically important due to its isolation from human sputum and various animals. In addition, previous studies by Kurtzman et al. [40] concluded that *Pichia* sp. is an important producer of the drug precursor 2-phenylethanol, used to manufacture antibiotics and other antimicrobial substances. Glucose uptake and ethanol productivity were evaluated for all the identified yeasts (Fig. 2). Although both tested strains were suitable to grow under glucose-enriched wastewater, only *Candida* sp. exhibited desirable fermentation properties as shown in Fig. 2a, b.

### Ethanol production from pretreated microalgae biomass

**Fig. 3:**
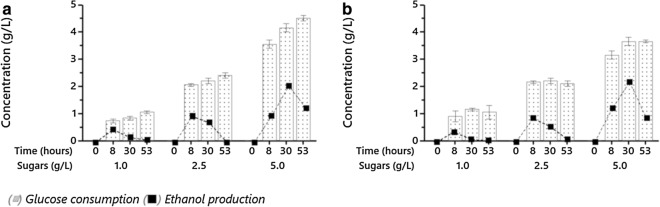
Glucose consumption and ethanol production during microalgal hydrolysate fermentation. **a** *Saccharomyces cerevisiae* S288C and **b** *Candida* sp

**Fig. 4:**
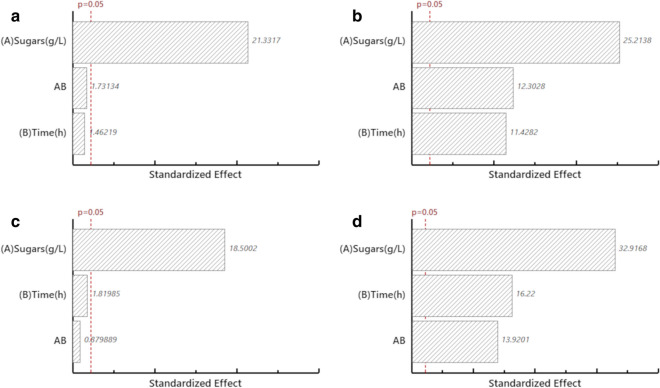
Effect of response variables during microalgal hydrolysate fermentation. *Saccharomyces cerevisiae* S288C: **a** Glucose consumption; **b** ethanol production. *Candida* sp.: **c** Glucose consumption; **d** ethanol production

Glucose consumption and ethanol production for *Saccharomyces cerevisiae* S288C and *Candida* sp. are shown in Fig. 3a and Fig. 3b, respectively. Similar glucose consumption rates were observed for both species after 8 h under all tested conditions. Factorial design effect analysis determined that *S. cerevisiae* S288C (Fig. 4a) and *Candida* sp. (Fig. 4c) exhibited significant differences in glucose consumption as the initial substrate concentration increased (*p* ≤ 0.05).

Chang et al. [43] reported similar results to this study, in which a *S. cerevisiae* strain gradually increased its consumption rate with glucose concentrations from 1 to 100 g L^−1^ in the fermentative media. The authors also reported not only a major slowdown at an initial substrate concentration of 150 g L^−1^, but also a significant inhibition of the alcoholic fermentation as the initial glucose concentration was increased up to 260 g L^−1^. This inhibitory effect was attributed to the osmotic effect caused by the high glucose concentrations, resulting in the slower proliferation of yeast cells, and ethanol production. Yet, the latter was not observed in this study, as glucose concentration was sufficient to maintain minimal osmotic stress conditions. Santos et al. [44] reported a similar glucose uptake rate to this study for *Candida utilis*, exhausting up to 88.9% of the glucose content in cachaça vinasse, which contained an initial glucose concentration of 3.6 g L^−1^.

Ethanol production for *S. cerevisiae* S288C (Fig. 4b) and *Candida* sp. (Fig. 4d) were significantly different as the initial glucose concentration and fermentation time increased (p ≤ 0.05). The results in this study indicated that the highest net ethanol production for both species was achieved at 8 h (1.0 and 2.5 g L^−1^) and 30 h (5.0 g L^−1^), respectively. Even though the ethanol productivity was low in comparison to other studies, mostly due to the relatively low initial substrate concentration, a marked decreased in the ethanol production was observed for all tested conditions. This might be attributed to diauxic growth, a condition where yeasts shift their pathway for energy production to an easily available substrate in order to maximize cell growth. According to Arroyo-López et al. [45] and de Smidt et al. [46], the ability to accumulate and consume ethanol is exclusive to *Saccharomyces* yeast due to a mutation in an alcohol dehydrogenase enzyme (ADH2) that benefits this genre over their competitors during fermentation, by first producing high ethanol levels and subsequently respiring it through the gluconeogenesis and glyoxylate cycle. The presence of this enzyme, especially in *S. cerevisiae*, is designated as one of the main reasons *Saccharomyces* strains are preferred as the principal microorganism for fermentation processes. This alcohol-reduction ability has recently been reported in non-*Saccharomyces* yeasts, e.g., *Candida*, *Kluyveromyces*, *Pichia*, *Dekkera*, etc., especially in the natural microflora present on grapes, harvesting and winemaking equipment [47]. However, the induction of this growth is not desirable at large-scale fermentation facilities and should be avoided to obtain higher ethanol efficiencies.

It is well known that fermentation efficiency is a key parameter for the industry. Thus, optimal conditions for higher ethanol productivity at a lower glucose consumption rate were assessed through Select Optimal Design tool in MINITAB®. Based on the obtained results from this analysis, it was determined that the best conditions were found in 5.0 g L^−1^ batches at a 30-h fermentation time. A summary of the parameters obtained for this condition in *S. cerevisiae* S288C and *Candida* sp. is shown in Table 2, as well as a comparison with similar species at slightly similar conditions.

## Conclusion

Among the different non-*Saccharomyces* yeast species isolated from a secondary wastewater effluent, only *Candida* sp. yeast species exhibited similar ethanologenic behavior to *S. cerevisiae* S288C. The identified yeast showed not only similar sugar consumption rates to *S. cerevisiae* but a near-to theoretical glucose-to-ethanol conversion yields (85%) at 5.0 g L^−1^ initial sugar concentrations. Moreover, alcohol consumption (reduction) as a growth substrate was also observed in *Candida* sp. which could be exploited in brewery industries for non-alcoholic beverages. Overall, this work provides an initial insight of free-living *Candida* strains as a fermenting microorganism in lab-scale reactors. Further studies are suggested to confirm its performance under higher glucose and ethanol concentrations, as well as its tolerance to other key parameters during fermentation such as pH, temperature, and dissolved oxygen.

## Materials and methods

### Wastewater characterization

The wastewater used in this study was obtained from the secondary settling tank of a municipal wastewater treatment plant located in Ciudad Universitaria, Mexico (19°19′14.6"N, 99°10′36.3"W) during the spring in 2018. Samples were taken in the surrounding turbulent flow area of the tank’s outlet pipe and stored in 20-L high-density polyethylene containers at 4.0 °C until use. In addition, any large, suspended solids were removed using 8.0-μm pore size Whatman™ glass microfiber filters (GE Healthcare Life Sciences, USA). Methods used to measure the wastewater physicochemical properties are shown in Table 1. All analyses were carried out in triplicates.

### Microalgae experimental setup

Microalgae cultivation was conducted using a previously adapted to wastewater microalgae inoculum, consisting mostly of *Scenedesmus* sp., provided by Laboratorio de Ingeniería Ambiental, Instituto de Ingeniería UNAM (Universidad Nacional Autónoma de México) as described by Oliveira et al. [52]. The microalgae inoculum was cultured in 5.0-L open-batch polyethylene terephthalate photobioreactors (height: 44.0 cm; ratio: 8.5 cm) with a maximum working volume of 80%. Operating conditions for each reactor used in this study were the following: manual aeration conditions (two times a day), temperature from 20 to 25 °C, and illumination provided by 20 W white light LED lamps (53 μmol m^−2^ s^−1^) with 12 h light and 12 dark cycles. Microalgae inoculum was directly added to wastewater inside the reactors until achieving an initial concentration of 300 ± 50 mgTSS L^−1^.

### Harvesting and pretreatment

After a 14-day period, microalgae biomass was harvested through centrifugation at 17,670 g using an Avanti® J-26S XPI High-Performance Centrifuge (Beckman Coulter Inc., USA) for 15 min at 5.0 °C. Then, the harvested microalgae slurry was recovered and dried using a BE-1449 Spray Dryer (Bowen Engineering Corp., USA) with an inlet and outlet temperature of 130 °C and 70 °C, respectively. The total sugar (carbohydrate) content was determined using the phenol–sulfuric acid colorimetric method as previously described in Table 3. Finally, dried microalgae biomass was collected and stored in polyethylene containers at room temperature inside an air-tight silica gel glass desiccator for subsequent sugar extraction and hydrolysis.

**Table 3:** Characterization of the secondary effluent from a municipal wastewater treatment plant used as culture medium. Analyses were performed in triplicate

Parameter (unit)	Concentration	Method	References
Temperature (°C)	26.6 ± 5.60	2550B	APHA-AWWA-WPCF [49]
pH	8.2 ± 0.45	4500-H^+^ B	
Turbidity (NTU)	6.7 ± 0.09	2130B	
Total suspended solids (TSS) (mg L^−1^)	26.7 ± 0.02	2540D	
Ammonia nitrogen (NH_3_-N) (mg L^−1^)	147.5 ± 1.32	4500-NH_3_ C	
Nitrates (NO_3_^−^-N) (mg L^−1^)	33.2 ± 8.72	8039	HACH Co. [50]
Phosphorus as orthophosphates (PO_4_^3−^-P) (mg L^−1^)	68.7 ± 2.68	8178	
Chemical oxygen demand (COD) (mg L^−1^)	121.0 ± 3.05	8000	
Carbohydrates (CHO) (mg L^−1^)	26.0 ± 1.67	Colorimetric	Hernández-García et al. [51]

For acidic pretreatment, microalgae biomass (20 gDW) was diluted in 200 mL of 1.0 M H_2_SO_4_ (Sigma-Aldrich, USA) and placed in a 250-mL round-bottom flask attached to a Liebig closed-condenser. The diluted biomass was heated to a temperature range of 85–90 °C and stirred at 1,200 rpm. Stirring speed and temperature were controlled using an RCT Basic Hotplate Magnetic Stirrer (IKA®, Germany) for 120 min. A 5.0 M solution of NaOH was used in order to adjust the pH of the medium to 6.5 ± 0.2 (neutralizer). Nonreactive solids and other impurities in the hydrolyzed medium were removed through centrifugation at 2,163 g for 20 min, pre- and post-neutralization. Finally, the sugar extraction (Eq. 1) and saccharification yield (Eq. 2) in the hydrolyzed medium were calculated as follows:

1 $$\% Ex = \frac{{Totalsugarconcentrationintheliquor(g/L)}}{{Initialbiomassload(g/L)*Sugarcontent(\% )}} \cdot 100$$

2 $$\% Sa = \frac{{Glucoseconcentrationintheliquor(g/L)*0.9}}{{Totalsugarconcentrationintheliquor(g/L)}} \cdot 100$$

where the factor 0.9 is referred to as the mass difference due to the monomer’s hydration after the hydrolysis. The sugar content of the residual slurry was measured using the phenol–sulfuric acid method as previously described. Glucose content in the hydrolyzed medium was verified through high-performance liquid chromatography (HPLC). Instrument and chromatographic conditions (Table S1), as well as the calibration curves for the tested monosaccharides (Fig S2), are listed in Additional file 1. All samples were filtered using 0.20-μm pore size sterilized membrane filters (Merck Millipore Co., Germany) and analyses were carried out in triplicates for each run.

### Identification and selection of ethanologenic yeast

In order to isolate potential ethanologenic wild-type yeast, wastewater samples were subjected to serial dilutions in sterilized water. Aliquots from each dilution were placed in yeast–peptone–dextrose (YPD) agar plates containing 1.0%w/v yeast extract, 2.0%w/v peptone, 2.0%w/v dextrose, and 150 μg mL^−1^ of chloramphenicol to inhibit bacterial growth. The plates were incubated for 48 h at 28 °C and colonies with uniform yeast-like morphology were sampled and streaked onto fresh YPD agar plates. An Axiolab.A1 microscope (Carl Zeiss, Germany) at × 100 magnification was used in order to observe the streaked colonies morphology and purity.

The isolated yeasts were identified by analyzing the Internal Transcribed Spacers (ITS) 1 and 2 sequencing patterns located between the 18S, 5.8S, and 28S rDNA subunits gene cluster. Yeast isolates were streaked onto yeast malt, YM, agar plates (Sigma-Aldrich, USA) and grown at 28 °C for 48 h. DNA was extracted from 500 mg of yeast biomass, using Fungal/Bacterial DNA MiniPrep™ kit (Zymo Research Corp., USA) according to the manufacturer’s instructions. The ITS regions were amplified from the extracted DNA by polymerase chain reaction (PCR) using universal primers ITS5 (5′-GGAAGTAAAAGTCGTAACAAGG-3′) and ITS4 (5′-TCCTCCGCTTATTGATATGC-3′) as previously described by White et al. [53]. Afterwards, the amplified regions were run on a 1.0% agarose gel at 100 V for 15 min in order to confirm a minimum fragment size of 500 bp. The resulting nucleotide sequences were edited using the trace viewer and editor software Chromas 2.4 to produce a single canonical sequence per isolated colony. Sequences were compared to the non-redundant sequence database at NCBI, National Center for Biotechnology Information, with BLAST (Basic Local Alignment Search Tool). BLAST search results were then aligned with the canonical sequence using the multi-sequence alignment program CLUSTALW and similarities were validated at a 99% query coverage and percentage identity.

Each successfully isolated yeast strain was tested for fermentation characteristics. First, batches of filtered wastewater were enriched with either 25 or 45 g L^−1^ of sterilized anhydrous d-dextrose (J.T. Baker, USA) and inoculated with yeast at an initial concentration of 0.100 OD_600_. Then, cultures were carried out inside an UNIMAX 1010 Orbital Incubator (Heidolph Instruments, Germany) at 28 °C and 180 RPM. Finally, parameters such as ethanol production and glucose consumption were measured using the HPLC method described in Additional file 1: Table S1. Samples were taken under aseptic conditions every three hours for 3 days. Replicates were not performed for the fermentation characteristics tests due to constant pore blockage of the membrane filters with bacteria and other microorganisms during wastewater filtration.

### Alcoholic fermentation assays

The resulting microalgae hydrolysate liquor was enriched with ammonia, nitrate and phosphorus sources prior to fermentation. Approximately 120.0 ± 5.2 mg L^−1^ of NH_4_Cl, 20.0 ± 7.61 mg L^−1^ of NaNO_3_, and 61.9 ± 2.51 mg L^−1^ of KH_2_PO_4_ were added to the hydrolysate in order to simulate the nutrient content commonly found in wastewater. Semi-batch fermentation experiments were performed in 50-mL conical polypropylene tubes (Fisher Scientific, UK) using two yeasts strains: *Saccharomyces cerevisiae* S288C (provided by Laboratorio de Genómica Facultad de Ciencias, UNAM) and the highest ethanol-producing yeast species from the previous section. As previously described, both species were first grown in YEPD broth and washed with sterilized distilled water preceding the inoculation.

All fermentation experimental runs were performed in duplicates, resulting in 18 experimental runs. The design matrix of the three-level factorial design is provided in the Additional file 1: Fig. S1. Ethanol production (X) and glucose consumption (Y) were selected as response variables. In contrast, fermentation time (A) and initial glucose concentration (B) were evaluated as independent factors as A affects the neat ethanol yield and acid content in fermenters while B is the limiting the fermentation reactions. “A” levels were selected at 8, 30 and 53 h based on preliminary experiments while “B” levels were established at 1.0, 2.5, 5.0 g L^−1^ due to the maximum extracted sugar content determined in the hydrolysate liquor.

The fermentations were inoculated with actively proliferating yeast cells in the enriched microalgae hydrolysate liquor at an OD_600_ of 0.100. For each tested condition, experimental blanks, consisting of sterilized nutrient-rich hydrolysate liquor, were also performed. All experiments were incubated at 28 °C and 120 RPM. Glucose consumption was measured using the previously described HPLC method while ethanol and acetic acid production were determined using the gas chromatography (GC) method and calibration curves described as Table S2 and Fig. S3, respectively, in the Additional file 1. The ethanol yield (Eq. 3) and productivity (Eq. 4) were determined as follows:

3 $$\mathrm{\%}EtOH=\frac{Ethanol produced (g {L}^{-1})}{0.511*initial sugar concentration (g {L}^{-1})}\cdot 100,$$

4 $$Productivity \left(\frac{g}{L h}\right)=\frac{\Delta Ethanol}{\Delta t},$$

where 0.511 is the maximum theoretical fraction of glucose-to-ethanol conversion according to the Gay-Lussac stoichiometry, Δ*Ethanol* is the difference between the initial and the final ethanol concentrations (ethanol produced), and Δ*t* is the time required to reach the maximum concentration value of ethanol. Prior to all measurements, yeast biomass was removed from the fermentation broth through centrifugation at 13,300 g for 5.0 min. All analyses were carried out in triplicates.

### Statistical analysis

The mean values and standard deviations reported in figures and tables were calculated using Microsoft Office Excel 2013. Statistical analyses were performed using MINITAB® 18 software. The significant differences among the results were analyzed through a three-level full factorial design followed by Tukey’s test (α = 0.05).

## Supplementary Information

**Additional file 1.** **Figure S1.** Design matrix with coded levels and real values (in parenthesis) for a three-level full factorial design. **Figure S2.** Calibration curve for tested parameters in HPLC using laboratory grade reagents. **Figure S3.** Calibration curve for ethanol quantification in GC using laboratory grade reagents. **Table S1.** High-Performance Liquid Chromatographer instruments and chromatographic conditions. **Table S2.** Gas Chromatographer instruments and chromatographic conditions.

## Data Availability

All data generated or analyzed during this study are included in this published article and its supplementary information files.

## Abbreviations

ADH2: Alcohol dehydrogenase enzyme
BLAST: Basic Local Alignment Search Tool
CHO: Carbohydrates
COD: Chemical oxygen demand
GC: Gas chromatograph
gDW: Grams of dried weight
HMFs: Hydroxymethylfurfurals
HPLC: High-performance liquid chromatography
ITS: Internal transcribed spacers
NCBI: National Center for Biotechnology Information
NH_3_-N: Ammonia nitrogen
NO_3_^−^-N: Nitrates
PCR: Polymerase chain reaction
PO_4_^3^^−^-P: Phosphorus as orthophosphates
TSS: Total suspended solids
YEPD: Yeast extract–peptone–dextrose
YM: Yeast malt
YPD: Yeast–peptone–dextrose

## References

[1] IEA. Key World Energy Statistics 2020. Paris: IEA Stat; 2020.

[2] Velazquez-Lucio J, Rodríguez-Jasso RM, Colla LM, Sáenz-Galindo A, Cervantes-Cisneros DE, Aguilar CN (2018). Microalgal biomass pretreatment for bioethanol production: a review. Biofuel Res J.

[3] Tobin T, Gustafson R, Bura R, Gough HL (2020). Integration of wastewater treatment into process design of lignocellulosic biorefineries for improved economic viability. Biotechnol Biofuels.

[4] Phwan CK, Chew KW, Sebayang AH, Ong HC, Ling TC, Malek MA (2019). Effects of acids pre-treatment on the microbial fermentation process for bioethanol production from microalgae. Biotechnol Biofuels.

[5] IEA. Key World Energy Statistics 2020. IEA Stat. 2020:80.

[6] Gielen D, Boshell F, Saygin D, Bazilian MD, Wagner N, Gorini R (2019). The role of renewable energy in the global energy transformation. Energy Strateg Rev.

[7] Pickl MJ (2019). The renewable energy strategies of oil majors—From oil to energy?. Energy Strateg Rev.

[8] Byreddy A, Gupta A, Barrow C, Puri M (2015). Comparison of cell disruption methods for improving lipid extraction from thraustochytrid strains. Mar Drugs.

[9] Sirajunnisa AR, Surendhiran D (2016). Algae–A quintessential and positive resource of bioethanol production: a comprehensive review. Renew Sustain Energy Rev..

[10] Venkata Mohan S, Rohit MV, Chiranjeevi P, Chandra R, Navaneeth B (2015). Heterotrophic microalgae cultivation to synergize biodiesel production with waste remediation: progress and perspectives. Bioresour Technol.

[11] de Farias Silva CE, Meneghello D, Bertucco A (2018). A systematic study regarding hydrolysis and ethanol fermentation from microalgal biomass. Biocatal Agric Biotechnol..

[12] Thangavelu SK, Ahmed AS, Ani FN (2016). Review on bioethanol as alternative fuel for spark ignition engines. Renew Sustain Energy Rev.

[13] Kumar D, Murthy GS (2011). Impact of pretreatment and downstream processing technologies on economics and energy in cellulosic ethanol production. Biotechnol Biofuels.

[14] Cuellar-Bermudez SP, Garcia-Perez JS, Rittmann BE, Parra-Saldivar R (2015). Photosynthetic bioenergy utilizing CO2: an approach on flue gases utilization for third generation biofuels. J Clean Prod.

[15] van Eijck J, Batidzirai B, Faaij A (2014). Current and future economic performance of first and second generation biofuels in developing countries. Appl Energy.

[16] Morone P, Cottoni L, Luque R, Lin CSK, Wilson K, Clark J (2016). Biofuels: technology, economics, and policy issues. Handbook of biofuels production.

[17] de Farias Silva CE, Bertucco A (2016). Bioethanol from microalgae and cyanobacteria: a review and technological outlook. Process Biochem.

[18] Behera S, Singh R, Arora R, Sharma NK, Shukla M, Kumar S (2015). Scope of algae as third generation biofuels. Front Bioeng Biotechnol..

[19] Miranda AF, Ramkumar N, Andriotis C, Höltkemeier T, Yasmin A, Rochfort S (2017). Applications of microalgal biofilms for wastewater treatment and bioenergy production. Biotechnol Biofuels.

[20] Ashokkumar V, Salam Z, Tiwari ON, Chinnasamy S, Mohammed S, Ani FN (2015). An integrated approach for biodiesel and bioethanol production from Scenedesmus bijugatus cultivated in a vertical tubular photobioreactor. Energy Convers Manag.

[21] Sun X-M, Ren L-J, Zhao Q-Y, Ji X-J, Huang H (2018). Microalgae for the production of lipid and carotenoids: a review with focus on stress regulation and adaptation. Biotechnol Biofuels.

[22] He M, Wu B, Qin H, Ruan Z, Tan F, Wang J (2014). Zymomonas mobilis: a novel platform for future biorefineries. Biotechnol Biofuels.

[23] Jiang Y, Wu R, Zhou J, He A, Xu J, Xin F (2019). Recent advances of biofuels and biochemicals production from sustainable resources using co-cultivation systems. Biotechnol Biofuels.

[24] Ruyters S, Mukherjee V, Verstrepen KJ, Thevelein JM, Willems KA, Lievens B (2015). Assessing the potential of wild yeasts for bioethanol production. J Ind Microbiol Biotechnol.

[25] Ali MN, Khan MM (2014). Screening, identification and characterization of alcohol tolerant potential bioethanol producing yeasts. Curr Res Microbiol Biotechnol.

[26] Tofighi A, Mazaheri Assadi M, Asadirad M, Zare KS (2014). Bio-ethanol production by a novel autochthonous thermo-tolerant yeast isolated from wastewater. J Environ Heal Sci Eng.

[27] Radecka D, Mukherjee V, Mateo RQ, Stojiljkovic M, Foulquié-Moreno MR, Thevelein JM (2015). Looking beyond Saccharomyces: the potential of non-conventional yeast species for desirable traits in bioethanol fermentation. FEMS Yeast Res.

[28] Holt S, Mukherjee V, Lievens B, Verstrepen KJ, Thevelein JM (2018). Bioflavoring by non-conventional yeasts in sequential beer fermentations. Food Microbiol.

[29] Serra Colomer M, Funch B, Forster J (2019). The raise of Brettanomyces yeast species for beer production. Curr Opin Biotechnol.

[30] Walls LE, Velasquez-Orta SB, Romero-Frasca E, Leary P, Yáñez-Noguez I, Orta Ledesma MT (2019). Non-sterile heterotrophic cultivation of native wastewater yeast and microalgae for integrated municipal wastewater treatment and bioethanol production. Biochem Eng J..

[31] Sanchez Rizza L, Sanz Smachetti ME, Do Nascimento M, Salerno GL, Curatti L (2017). Bioprospecting for native microalgae as an alternative source of sugars for the production of bioethanol. Algal Res.

[32] Borowitzka MA, Borowitzka MA, Moheimani NR (2013). Techno-economic modeling for biofuels from microalgae. Algae for biofuels and energy.

[33] Padmaperuma G, Kapoore RV, Gilmour DJ, Vaidyanathan S (2018). Microbial consortia: a critical look at microalgae co-cultures for enhanced biomanufacturing. Crit Rev Biotechnol.

[34] Bastos RG, Jacob-Lopes E, Queiroz Zepka L, Queiroz MI (2018). Biofuels from microalgae: bioethanol. Energy from microalgae.

[35] de Farias Silva CE, Meneghello D, de Souza Abud AK, Bertucco A (2018). Pretreatment of microalgal biomass to improve the enzymatic hydrolysis of carbohydrates by ultrasonication: Yield vs energy consumption. J King Saud Univ Sci..

[36] Valdez-Guzmán BE, Rios-Del Toro EE, Cardenas-López RL, Méndez-Acosta HO, González-Álvarez V, Arreola-Vargas J (2019). Enhancing biohydrogen production from *Agave tequilana bagasse*: Detoxified vs Undetoxified acid hydrolysates. Bioresour Technol.

[37] Seon G, Kim HS, Cho JM, Kim M, Park WK, Chang YK (2020). Effect of post-treatment process of microalgal hydrolysate on bioethanol production. Sci Rep.

[38] Castro YA, Ellis JT, Miller CD, Sims RC (2015). Optimization of wastewater microalgae saccharification using dilute acid hydrolysis for acetone, butanol, and ethanol fermentation. Appl Energy.

[39] Kurtzman CP, Fell JW, Boekhout T. The yeasts: a taxonomic study. Amsterdam: Elsevier; 2011.

[40] Miranda JR, Passarinho PC, Gouveia L (2012). Pre-treatment optimization of Scenedesmus obliquus microalga for bioethanol production. Bioresour Technol.

[41] Kieliszek M, Kot AM, Bzducha-Wróbel A, Łażejak BS, Gientka I, Kurcz A. Biotechnological use of Candida yeasts in the food industry: a review. Fungal Biol Rev. 2017;31:185–98. https://www-sciencedirect-com.libproxy.ncl.ac.uk/science/article/pii/S1749461317300350. Accessed 10 Oct 2018.

[42] Kieliszek M, Błażejak S (2016). Current knowledge on the importance of selenium in food for living organisms: a review. Molecules.

[43] Chang Y-H, Chang K-S, Chen C-Y, Hsu C-L, Chang T-C, Jang H-D (2018). Enhancement of the efficiency of bioethanol production by saccharomyces cerevisiae via gradually batch-wise and fed-batch increasing the glucose concentration. Fermentation.

[44] dos Santos JF, Canettieri EV, Souza SMA, Rodrigues RCLB, Martínez EA (2019). Treatment of sugarcane vinasse from cachaça production for the obtainment of Candida utilis CCT 3469 biomass. Biochem Eng J.

[45] de Smidt O, Du Preez JC, Albertyn J. The alcohol dehydrogenases of Saccharomyces cerevisiae: A comprehensive review. FEMS Yeast Res. 2008;8:967–78. 10.1111/j.1567-1364.2008.00387.x 18479436

[46] Arroyo-López FN, Salvadó Z, Tronchoni J, Guillamón JM, Barrio E, Querol A (2010). Susceptibility and resistance to ethanol in Saccharomyces strains isolated from wild and fermentative environments. Yeast.

[47] Contreras A, Hidalgo C, Henschke PA, Chambers PJ, Curtin C, Varela C (2014). Evaluation of non-Saccharomyces yeasts for the reduction of alcohol content in wine. Appl Environ Microbiol.

[48] Kallarakkal KP, Muthukumar K, Alagarsamy A, Pugazhendhi A, Naina MS (2021). Enhancement of biobutanol production using mixotrophic culture of *Oscillatoria* sp. in cheese whey water. Fuel.

[49] APHA-AWWA-WPCF (2005). Standard methods for the examination of water and wastewater.

[50] HACH Co (2000). Manual de análisis de agua.

[51] Hernández-García A, Velásquez-Orta SB, Novelo E, Yáñez-Noguez I, Monje-Ramírez I, Orta Ledesma MT (2019). Wastewater-leachate treatment by microalgae: biomass, carbohydrate and lipid production. Ecotoxicol Environ Saf.

[52] Oliveira GA, Carissimi E, Monje-Ramírez I, Velasquez-Orta SB, Rodrigues RT, Ledesma MTO (2018). Comparison between coagulation-flocculation and ozone-flotation for *Scenedesmus microalgal* biomolecule recovery and nutrient removal from wastewater in a high-rate algal pond. Bioresour Technol.

[53] White TJ, Bruns T, Lee S, Taylor J. Amplification and direct sequencing of fungal ribosomal RNA genes for phylogenetics. In: PCR Protocols. 2014. p. 315–22.

