# Supplementary material for: Bioprospecting of wild type ethanologenic yeast for ethanol fuel production from wastewater-grown microalgae
Source: Biotechnol Biofuels. 2021 Apr 9;14:93. doi: 10.1186/s13068-021-01925-x (PMC8035739; doi:10.1186/s13068-021-01925-x)
Supplement: Supplementary file 1 — Additional file 1. Figure S1. Design matrix with coded levels and real values (in parenthesis) for a three-level full factorial design. Figure S2. Calibration curve for tested parameters in HPLC using laboratory grade reagents. Figure S3. Calibration curve for ethanol quantification in GC using laboratory grade reagents. Table S1. High-Performance Liquid Chromatographer instruments and chromatographic conditions. Table S2. Gas Chromatographer instruments and chromatographic conditions. [file 13068_2021_1925_MOESM1_ESM.docx]

Supplementary information

*Fig S1. Design matrix with coded levels and real values (in parenthesis) for a three-level full factorial design*

| **Run** | **Coded value** | | **Real values** | |
| --- | --- | --- | --- | --- |
|  | A | B | A: Time (h) | B: Glucose concentration (g L^-1^) |
| 1 | +1 | - 1 | 8.0 | 5.0 |
| 2 | 0 | - 1 | 8.0 | 2.5 |
| 3 | - 1 | - 1 | 8.0 | 1.0 |
| 4 | 0 | +1 | 53.0 | 2.5 |
| 5 | - 1 | +1 | 53.0 | 1.0 |
| 6 | +1 | 0 | 30.0 | 5.0 |
| 7 | 0 | +1 | 53.0 | 2.5 |
| 8 | - 1 | - 1 | 8.0 | 1.0 |
| 9 | +1 | +1 | 53.0 | 5.0 |
| 10 | +1 | 0 | 30.0 | 5.0 |
| 11 | 0 | 0 | 30.0 | 2.5 |
| 12 | - 1 | 0 | 30.0 | 1.0 |
| 13 | +1 | - 1 | 8.0 | 5.0 |
| 14 | - 1 | +1 | 53.0 | 1.0 |
| 15 | - 1 | 0 | 30.0 | 1.0 |
| 16 | 0 | 0 | 30.0 | 2.5 |
| 17 | 0 | - 1 | 8.0 | 2.5 |
| 18 | +1 | +1 | 53.0 | 5.0 |

*Fig S2. Calibration curve for tested parameters in HPLC using laboratory grade reagents*

*Fig S3. Calibration curve for ethanol quantification in GC using laboratory grade reagents*

*Table S1. High-Performance Liquid Chromatographer instruments and chromatographic conditions*

| **Instrument** | Perkin Elmer® Flexar™ LC |
| --- | --- |
| **Column** | Restek^TM^ Ultra Amino  (150 mm column length , 4.6 mm inside diameter, 3.0 μm particle size, 100 A° pore size) |
| **Injector** | Rheodyne  (20 μL loop) |
| **Injection volume (μL)** | 20 |
| **Mobile phase** | Acetonitrile (70%): Water (30%) |
| **Carrier flow (mL min^-1^)** | 0.8 |
| **Detector temperature (°C)** | 35 |
| **Detector** | Refractive Index (RI)  Water 410 |

*Table S2. Gas Chromatographer instruments and chromatographic conditions*

| **Instrument** | Shimadzu^®^ GC 2010plus |
| --- | --- |
| **Column** | GC Zebron^®^ ZB-WAXplus^™^  (30 m length, 0.26 mm internal diameter, 0.25 μm film thickness) |
| **Injector** | Shimadzu^®^ AOC 20i  (1.0 μL Loop) |
| **Injection volume (μL)** | 1.0 |
| **Mobile phase** | Ultrapure Helium (He) ≥ 99.0% |
| **Carrier pressure (psi)** | 21.2 |
| **Carrier flow (mL min^-1^)** | 2.0 |
| **Column oven temperature (°C)** | 250 |
| **Rate of temperature increase (Ramp)**  **(°C min^-1^)** | 50 |
| **Detector temperature (°C)** | 50 |
| **Detector** | Barrier discharge ionization detector (BID 2010plus) |
